# Women’s autonomy in health decision-making and its effect on access to family planning services in Senegal in 2017: a propensity score analysis

**DOI:** 10.1186/s12889-020-09003-x

**Published:** 2020-06-05

**Authors:** N. M. Sougou, O. Bassoum, A. Faye, M. M. M. Leye

**Affiliations:** 1 Department of Preventive Medicine and Public Health, University of Cheikh Anta Diop, Dakar, Senegal; 2 Institute of Health Development, University of Cheikh Anta Diop, Dakar, Senegal; 3 UMI 3189 « Environnement, Santé, Sociétés », UCAD, CNRS, CNRST, USTTB, UGB, Dakar, Senegal

**Keywords:** Autonomy of decision-making for health, Family planning, Senegal

## Abstract

**Background:**

The effect of women’s autonomy in decision-making for fertility control has been highlighted by research. The objective of this study was to analyze the effect of women’s autonomy over decision-making regarding their health and access to family planning in Senegal in 2017.

**Methods:**

The analyses in this study were carried out using data from the Senegal Demographic and Health Survey in 2017. The sample consisted of 8865 women aged 15–49. The propensity score-matching method was applied. Autonomy in health decision-making was considered the treatment variable. Matching was performed using confounding variables. The outcome variables were the current use of modern contraceptive methods and the existence of unmet needs. The common support condition had been met. The analysis was conducted using STATA.15 software.

**Results:**

This study showed that 6.26% of women had decision-making autonomy in relation to their health. For 80.33% of the women, their husbands/partners made health-related decisions for them. Decision-making autonomy increased significantly with the age of the woman (*p* < 0.05). In addition, 15.24% of women were using a modern method of contraception. An estimated 26.2% of women had unmet needs. Propensity score matching split the women into two groups based on autonomy over decision-making for their health. After matching, there was no longer a significant difference between women who were autonomous with respect to their decision-making and those who were not autonomous with respect to their current use of a modern contraceptive method. On the other hand, there was a 14.42% reduction (*p* < 0.05) in unmet needs for family planning in the group of women who were autonomous with respect to their health decision-making.

**Conclusion:**

Autonomy in health decision-making would reduce unmet needs among Senegalese women. These results show the importance of accounting for gender in health interventions for the accessibility of family planning services.

## Background

In 2015, 303,000 women died during or after pregnancy or childbirth. Most of these deaths occurred in low-income countries, and most of them could have been prevented [1]. Access to contraception has been described as one of the key interventions to combat maternal mortality. In 2017, 63% of women worldwide were using some form of contraception. Contraceptive use was above 70% in Europe, Latin America, the Caribbean, and Northern America, while it was low (at 25%) in Central and Western Africa [2]. In Senegal, despite the implementation of numerous health interventions, the use of modern contraceptive methods among couples remained low, with a contraceptive prevalence rate of 18.9% and a high rate of unmet needs for family planning (25.2%) [3]. In Senegal, progress in family planning has long been reflected in a gradual increase in women’s current use of modern contraceptive methods, while the unmet need rate remains high [4]. The latter represent women who either wish to avoid all pregnancies or to space out or limit future pregnancies and yet do not use modern contraception [5]. According to some studies, the reasons for these unmet needs are related to access to family planning services on the one hand and to the behavioral characteristics of women and their partners on the other hand [6].

Some studies had already focused on identifying factors associated with the use of family planning services. The determinants identified were factors related to sociodemographic characteristics (place of residence, women’s level of education, socioeconomic level), factors related to the health system (insufficient family planning services) and sociocultural factors [7]. However, few studies have focused on gender relations within family and social structures and their implications for contraceptive practices. Nevertheless, in some African countries, contraceptive knowledge and practices were influenced by sociocultural norms such as the domination of men and husbands, opposition to contraception, and the lower social status of women [8]. Women’s participation in fertility decision-making has been described as a determinant of contraceptive practices [7]. Recent research and policy discourse generally view the limited autonomy of women in developing countries as a major obstacle to improving their reproductive health [9–11]. The objective of this study was to investigate the relationship between women’s autonomy over health decision-making and family planning. This study will make it possible to show the involvement of gender relations in the reduction of unmet needs for contraception in Senegal.

## Methods

### Type of study

The study was cross-sectional, descriptive and analytical.

### Data

This study consisted of a secondary analysis of data from the Senegal Demographic and Health Survey (DHS) 2017, a nationally representative survey of 8865 women aged 15–49. The sample from the 2017 Continuous DHS is representative at the national level, at the regional level, and for both urban and rural areas [12]. The sample was drawn stratum by stratum. Thus, the sample is based on a stratified, two-stage area survey. At the first stage, 400 clusters (primary survey units) were drawn from the list of enumeration areas established during the General Census of Population and Housing, Agriculture and Livestock conducted in 2013, using a systematic draw with probability proportional to size, the size of the primary survey units being the number of households. A count of households in each of these clusters provided a list of households from which a sample of 22 households per cluster were drawn at the second stage with equal probability systematic sampling in both urban and rural areas. A total of 8800 households (4092 urban and 4708 rural) were selected.

The survey included detailed information on fertility, nuptiality, sexual activity, fertility preferences, knowledge and use of family planning methods. Data were collected using four questionnaires: (i) the household questionnaire, (ii) the women’s questionnaire, (iii) the men’s questionnaire, and (iv) the biomarker questionnaire. These four collection tools are based on the DHS Program’s model questionnaires and have been adapted with respect to Senegal’s demographic and health context.

The individual record (IR) file was used for the analysis. This female individual questionnaire was used to record information from women aged 15–49 who were residents or visitors the night before the survey.

The explanatory variable was women’s autonomy over decision-making for their health. This variable was generated from the DHS variable “person who usually decides on respondent’s health care (women)”. This variable had 5 modalities: the respondent herself, the respondent and her husband/partner, the husband/partner alone, someone else and others. The variable was recoded as a binary variable. This variable was binary and coded 1 (has autonomy over decision-making for her health) and 0 (does not have autonomy over decision-making for her health). The outcome variables were contraceptive use and the existence of unmet needs.

The outcome variable “contraceptive use” was a binary variable coded as “1” (currently uses a contraceptive method) and “0” (does not use a contraceptive method). The outcome variable “existence of unmet need” was binary with 2 modalities: “existence of an unmet need for contraception” (coded 1) and “no unmet need for contraception” (coded 0).

The matching variables in this analysis were those that had been identified as potentially confounding the association between decision-making power and the existence of unmet needs in this subpopulation of women. The individual factors included the following: (1) age was analyzed by 4-year age groups (15–19 years; 20–24 years; 25–29 years; 30–34 years; 35–39 years; 40–44 years; and 45–49 years); (2) women’s marital status was classified as “never in a union”, “married”, “in a union”, “widowed”, “separated” and “divorced”; and (3) women’s educational level was classified as “uneducated”, “primary”, “secondary” and “tertiary”. The household factors included the following: (1) household wealth - the wealth index, a measure of relative economic well-being based on household assets, was classified into quintiles (lowest, second, middle, fourth, highest) and was derived from the wealth score; and (2) place of residence was dichotomized into “urban” or “rural”.

### Analysis

Data were explored, and frequencies and cross-tabulations were generated to clarify the distribution patterns of the variables of interest. Tests of comparisons of means were performed on the variables of interest in relation to decision-making power.

Potential selection biases were corrected by establishing a propensity score for women’s exposure to decision-making autonomy (see Table 1).

**Table 1:** Distribution of variables according to access to health or non-health decision making before and after matching

	Autonomy in relation to the decision for their health					
	Before pairing			After pairing		
	Yes	No	p	Yes	No	p
	%	%		%	%	
Age (under 19 years)	36.79	10.07	0.0003	36.79	36.79	1
Place of residence	15.55	16.97	0	15.55	15.55	0.96
Women’s level of education	55.85	45.62	0.02	55.85	53.11	0.71
Level of poverty	27.95	24.61	0	27.96	28.03	0.91
Marital status	96.32	99.50	0	96.32	96.32	1

After testing the common support hypothesis, the propensity score was calculated for all women using a multivariate logistic regression that included the covariates elicited. The propensity scores thus obtained, i.e., the conditional probability of women being able to independently make decisions regarding their health, allowed for matching between two groups. Propensity score analysis is a method of adjustment that consists of deriving the conditional probability (the propensity score) of a patient to receive the treatment being evaluated, knowing her characteristics measured upon inclusion. Matching each treated subject to an untreated subject with an identical or close propensity score results in the formation of two groups of subjects with comparable characteristics and between whom the criterion of judgment can be compared [13].

We used the STATA PSMATCH2 module of STATA.15 software to perform 5:1 matching [14]. We used the neighbors function (5).

The outcome variables were “contraceptive use” and “unmet need”. Simple logistic regression models were constructed to examine the unadjusted and adjusted effects of women’s decision-making power on the current use of modern contraceptives and the existence of unmet needs among Senegalese women aged 15–49.

## Results

### Participants

In our study, a secondary analysis of the Senegal 2017 DHS data was performed. Participants from urban and rural areas were selected from all 14 administrative regions of Senegal. The study focused on health decision-making among women aged 15–49.

**Fig. 1:**
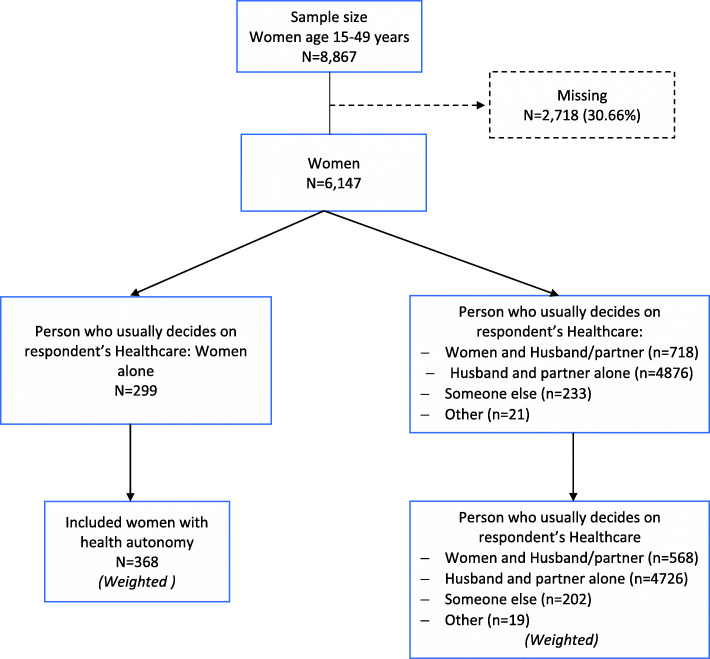
Diagram flow of the population of study

After the issue of missing responses was addressed, the population size for our study was 5147 women. The flow diagram of the study population is represented in Fig. 1.

### Characteristics of women

Sociodemographic, economic and behavioral characteristics among women aged 15–49.

A total of 66.5% of women were uneducated (see Table 2).

**Table 2:** Distribution of socio-demographic, economic and behavioral characteristics among women aged 15–49 (weighted) [Senegal DHS, 2017]

	Freq	Percent %	Missing
Age			
15–19	1998	22.5	
29–24	1664	18.77	
25–29	1529	17.19	
30–34	1318	14.87	
35–39	998	11,26	
40–44	806	9.09	
45–49	557	6.28	
Place of residence			
Rural	4514	50.92	
Urban	4351	49.08	
Level of education			
Uneducated	4310	48.62	
Primary level	1967	22.19	
Secondary level	2241	25.28	
Higher level	347	3.91	
Level of education of husband/partner			2339 (26.38%)
Uneducated	4073	66.55	
Primary level	607	9.62	
Secondary level	584	9.25	
Higher level	297	4.70	
Don’t know	750	11,88	
Wealth index			
the poorest	1475	16.64	
the poor	1603	18.08	
the middle	1749	19.73	
the rich	1991	22.46	
the richest	2046	23.08	
Marital status			
Married	3047	34.37	
Not Married	5818	65.63	

Women’s autonomy over decision-making.

The study showed that 6.26% of women had the latitude make their own decisions about their health, while for 80.33%, their husbands or partners made such decisions for them (see Table 3).

**Table 3:** Distribution of the person who decides for women’s health according to these characteristics (*N* = 5883)

Person who usually decides for woman’s health Workforce.	Frequency	Percentage
Woman herself	368	6.26
Woman in consultation with husband/partner	568	9.66
Husband/partner alone	4726	80.33
Someone else	202	3.43
Other	19	0.32

**Fig. 2:**
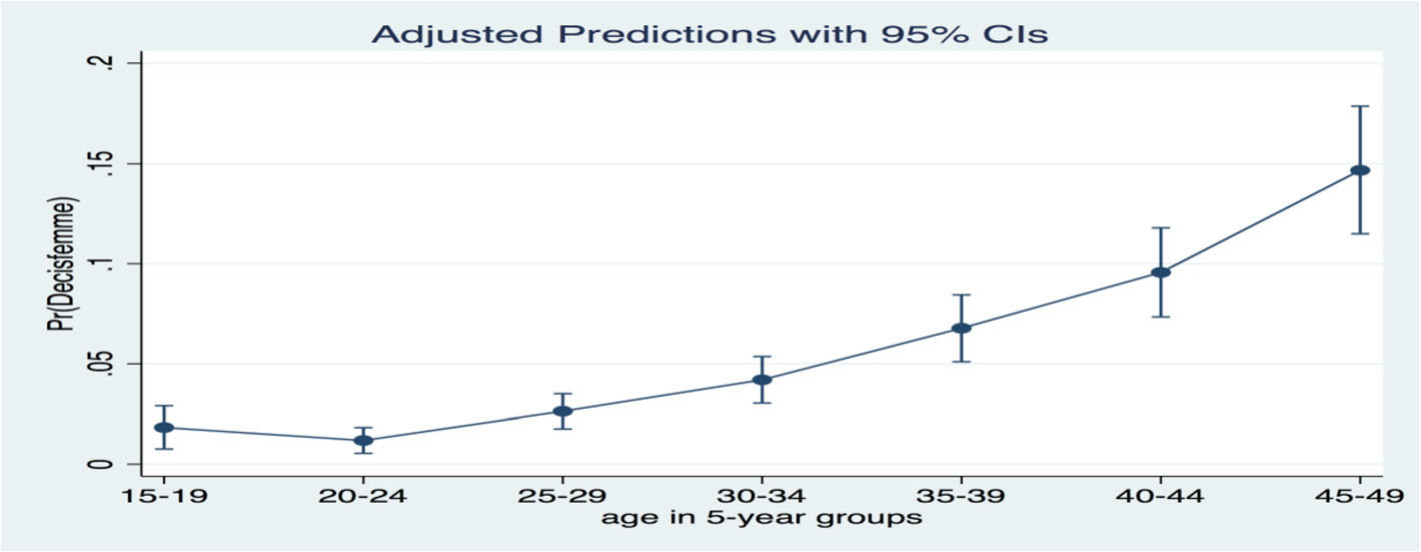
Women’s access to decision-making about their health according to their age

Women’s autonomy over decision-making increases with age (see Fig. 2).

Women living in rural areas have less autonomy over decision-making for their health (OR: 0.54[0.43–0.68]). Women with a higher level of education are 5.5 times more likely to have autonomy over decision-making. Richer and wealthier women have more autonomy over decision-making, with ORs of 2.74 [1.59–4.70] and 2.88 [1.73–4.79], respectively. Women in unions (cohabitation) have more autonomy over decision-making. Women whose partners/husbands are educated have more autonomy over decision-making for their health (OR: 2.49 [1.37–4.52] and OR: 2.66 [1.35–5.24] for primary and secondary education of partners/husbands, respectively). Educated women also have more autonomy over decision-making (OR: 5.52 [3.06–9.94]) (see Table 4).

**Table 4:** Factors associated with women’s decision-making autonomy

Women’s access to decision-making	Percentage %	p	OR	CI 95%
Place of residence		0		
Rural	2.23		0.54	0.43–0.68
Urban	4.03		1	
Level of education		0.003		
Uneducated	3.43		1	
Primary level	1.32		1.08	0.66–1.8
Secondary level	0.85		1.19	0.57–2.47
Higher level	0.66		5.52	3.06–9.94
Level of education of husband/partner		0.033		
Uneducated	64.55		1	
Primary level	9.62		2.49	1.37–4.52
Secondary level	9.25		2.66	1.35–5.24
Higher level	4.7		1.92	0.88–4.16
Don’t know	11.88		0.97	0.55–1.72
Wealth index		0.0007		
the poorest	0.71		1	
the poor	1.17		1.77	1.07–2.94
the middle	0.85		1.35	0,83–2.21
the rich	1.77		2.74	1.59–4.70
the richest	1.75		2.88	1.73–4.79
Marital status		0.0001		
Married	5.91		1	
In union	1.1		7.17	2.36–21.74

#### Contraceptive use

In this study, contraceptive prevalence was estimated at 17.9% of women in Senegal.

The most commonly used modern contraceptive methods are injectables (5.75%), implants (5.21%) and pills (3.11%) (see Table 5).

**Table 5:** Distribution of women according to the type of contraceptive method used

Current use of contraceptive method	Percentage %	SE
Does not use a method	82.04	0.69
Pills	3.11	0.28
IUD	1.17	0.19
Injectables	5.75	0.31
condom	0.63	0.14
female sterilization	0.5	0.14
Abstinence	0.54	0.13
Coil interrupted	0.29	0.08
Other methods	0.56	0.1
implants/norplant	5.21	0.45
LAM	0.01	0.03
collar	0.04	0.02

In this study, the prevalence of women with unmet needs was 26.2%.

Among women using contraception, 2.14% have autonomy over decision-making for their health. Among women with unmet needs, only 0.09% have autonomy over decision-making for their health.

Propensity score adjustment.

Unmet needs were reduced by 14.42% in the group of women with autonomy over health decisions (*p* < 0.05). There were no differences in contraceptive use between the 2 groups of women (Table 6).

**Table 6:** Effects of access to decision-making power on contraceptive use and unmet need among women after adjustment by propensity score

	Autonomy in relation to the decision							
	Before pairing				After matching			
	Yes	No	Difference	p	Yes	No	Difference	p
	%	%			%	%		
Existence of unmet needs	16.39	23.8	7.42	0.0032	16.44	30.87	14.43	0.001
Contraceptive use	29.43	21.36	−8.07	0.001	29.53	21.54	−7.99	0.06

## Discussion

### Factors associated with decision-making autonomy among Senegalese women

In Africa, the family environment is the locus of the relationship of domination between the sexes, the aim of which remains the subordination of gender [15]. Studies on gender and development have made it possible to highlight the contribution of “empowerment”, or access to autonomy, which is at the heart of the resolution of social inequalities linked to gender [16].

In Senegal, women’s autonomy over decision-making for their health is still weak. Our study has shown that only 6.26% of women have the latitude to make decisions regarding their own health. For most women (80.33%), their husbands or partners decide for them. Elsewhere, in several developing countries, studies have shown how certain cultural norms affect women’s autonomy in making decisions about their health [17]. It is mainly about the domination of the husband/partner, who often makes decisions for women on health issues [18].

This study shows that women’s power to make their own health-related decisions increases with their age. Other studies have shown the influence of age on women’s empowerment [19]. In other countries in Africa, few women are empowered to make decisions about their own health [20]. Several studies in developing countries have also shown the positive influence of women’s advanced age on their access to autonomy in decision-making about their health [21]. The older a woman becomes in traditional African societies, the more autonomous she becomes. The social construction of women’s position and their position changes according to their age and their role in society [22]. However, in various traditional societies, women may appear to be important, influential or even autonomous, but compared to men of the same age, their positions generally suffer from a lack of recognition and value [23].

Our study showed that women in the richest and wealthiest quintiles have more autonomy over decision-making. In Ethiopia, a study on access to decision-making autonomy for health reached similar conclusions. Women with a high socioeconomic level had better access to decision-making autonomy over their health [24].

One of the factors associated with decision-making autonomy is educational attainment. Thus, both the educational level of the woman and that of her partner are factors associated with women’s decision-making autonomy. In our study, the highest level of education is associated with decision-making autonomy in relation to health. In Nepal, women’s higher education was positively associated with autonomy in healthcare decision-making (*p* < 0.01), but their higher education was not significant relative to other outcome measures [25]. In other countries, increasing women’s autonomy has been shown to be positively correlated with women’s education [26].

In our study, women with educated partners/husbands had greater autonomy over decision-making for their health. These are generally men with primary and secondary education. Studies have shown that well-educated men tend to have fewer sexist ideologies [18]. Educating men is all the more important, as support from the partner and the family environment has been shown to have a positive impact on strengthening women’s decision-making autonomy [27].

With low levels of education in the community, rural areas have shown limitations in supporting women. Studies have shown that family structure and gender relations in rural areas can influence women’s autonomy [28] . In this study, women living in rural areas have less access to decision-making autonomy over their health. In Africa, patriarchy is often more developed in rural regions [29]. In addition, sociocultural burdens are more pronounced in rural areas [30].

In Senegal, despite numerous health interventions, the unmet need for contraception remains high. At the same time, the prevalence of contraceptives has increased in recent years [31]. This study showed an unmet need rate of 26.2% among Senegalese women. The persistence of this unmet need for contraception suggests that health interventions have not yet addressed the determinants that keep the unmet need high. The study showed that autonomous decision-making for health reduces the rate of unmet needs by 14% (*p* < 0.05). Thus, this study shows that women who continue to have unmet needs are women who are not autonomous in making decisions for their own health. For most (80.33%), the primary decision-maker with respect to their health is their husbands.

Thus, this study highlights the importance of studying gender relations in understanding the factors limiting access to sexual and reproductive services. Elsewhere, in other developing countries, studies have shown how certain cultural norms affect women’s autonomy in making decisions about their health [17, 18]. These include, as in this study, the dominance of the husband/partner makes decisions for the woman in matters related to her health.

In addition, our study did not highlight a significant link between the use of a contraceptive method and decision-making power. On the other hand, the analysis of data from the DHS carried out in 32 countries in sub-Saharan Africa has shown a significant relationship between these two variables [32]. The methodological differences in terms of statistical analyses could explain these contradictory results.

### Limitations

One of the major strengths of this study is the use of propensity scores, which eliminated the impact of selection bias inherent in observational studies (as opposed to randomized clinical trials, in which the principle is to achieve initial group comparability). This statistical method resulted in two groups that were comparable overall except for the explanatory variable (autonomy in decision-making). For statistical analysis, we used the STATA PSMATCH2 module of STATA.15 software to perform 5:1 matching. From a statistical point of view, some limitations can be pointed out regarding the use of the PSMATCH2 command rather than TEFFETCS. The STATA PSMATCH2 command has some advantages, as it allows the PSTEST command to be used to evaluate the comparability of the treatment and control groups according to the covariates specified before and after matching. However, the latter has some limitations that could have been considered by the TEFFECTS PSMATCH command. Indeed, with PSMATCH2, there is an insufficiency in the consideration of standard errors when calculating the propensity score.

Another strength of this study lies in the use of DHS data from Senegal, which are representative of Senegal. This makes it possible to generalize the results of the study to women of childbearing age in Senegal. However, this study has certain limitations. In particular, the analyses used cross-sectional data, so that only associations and no causal relationships were established. This study could be complemented by a qualitative study to clarify the social contexts in which gender relations evolve.

## Conclusion

Based on the findings of this study, it is imperative to note that improving women’s autonomy in decision-making for their health could contribute to the reduction of unmet needs. This study showed that women’s empowerment for their health is associated with other immediate determinants, including their age, marital status, education, partner’s education, living environment (rural/urban), wealth or socioeconomic status. Improving women’s participation in the labor force, which entails creating employment opportunities, reducing gender-based violence, strengthening their decision-making power and increasing their level of knowledge, could contribute to better access to family planning services and, above all, to reducing unmet needs, thus improving maternal health in Senegal. In addition, the development of transformative health programs would provide better access to sexual and reproductive health services for women. One of the recommendations of this study would be to promote the establishment of multisectoral collaboration in an integrated family planning program in Senegal. This multisectorality could include the education sector with interventions in favor of education and literacy for women and the microfinance and entrepreneurship sector in the context of the promotion of income-generating activities. In addition, the results of this study suggest that men should be enlisted in family planning awareness programs.

## Data Availability

The datasets analyzed during the current study are publicly available upon request at https://dhsprogram.com/data/available-datasets.cfm.

## Abbreviations

OR: Odds ratio
CI: Confidence intervals
DHS: Demographic Health Survey
SE: Standard error; National Ethics Committee (CNERS)
LAM: Lactational amenorrhea method
IR: Individual record
